# Intestinal Epithelial Cell-Related Alternative Splicing Events in Dextran Sodium Sulfate-Induced Acute Colitis

**DOI:** 10.5152/tjg.2023.22572

**Published:** 2023-05-01

**Authors:** Xiaoning Lin, Bijuan Zheng, Feiteng Gu, Xin Fan, Jianlin Shen

**Affiliations:** 1 Department of Orthopedics, Affiliated Hospital of Putian University, Putian, Fujian, China; 2 Department of Gastroenterological Surgery, Affiliated Hospital of Putian University, Putian, Fujian, China; 3 Department of Pediatrics, Affiliated Hospital of Putian University, Putian, Putian, China; 4 Chongqing Academy of Animal Sciences, Chongqing, China; 5 Department of General Surgery, Xinqiao Hospital, Army Medical University, Chongqing, China

**Keywords:** Inflammatory bowel disease,, intestinal epithelial cells,, alternative splicing,, replicate Multivariate Analysis of Transcript Splicing,, apoptotic

## Abstract

**Background:**

Alternative splicing of pre-messenger RNA is recognized as the crucial mechanism for gene expression regulation and proteome diversity generation. Alternative splicing has been found to be related to the pathogenesis of inflammatory bowel disease. The aim of this study was to identify the alternative splicing events in intestinal epithelial cells from mouse models of acute colitis and expand the understanding of the pathogenesis of inflammatory bowel disease.

**Methods:**

The acute colitis mouse models were constructed, and intestinal epithelial cells of the colon were isolated for RNA sequence. The replicate Multivariate Analysis of Transcript Splicing software was used to analyze the alternative splicing events. The functional analysis was performed on genes with significant differential alternative splicing events. The alternative splicing events of picked genes were validated by reverse transcription polymerase chain reaction.

**Results:**

A total of 340 significant differential alternative splicing events (from 293 genes) were screened out in acute colitis, and the alternative splicing events of CDK5-regulatory subunit associated protein 3 and TRM5 tRNA methyltransferase 5 were validated. The functional analysis showed that differential alternative splicing events in acute colitis participate in the apoptotic process, and the alternative splicing events of 3 genes (BCL2/adenovirus E1B-interacting protein 2, tumor necrosis factor receptor-associated factor 1, and tumor necrosis factor receptor-associated factor 7) were validated by reverse transcription polymerase chain reaction.

**Conclusion:**

This study pointed out the potential impact of different alternative splicing in acute colitis.

## INTRODUCTION

Inflammatory bowel disease (IBD) is a common gastrointestinal tract disorder that usually comes in 2 types: ulcerative colitis (UC) and Crohn’s disease (CD). The occurrence of IBD may be related to environmental factors, genetic factors, or infectious factors; however, the exact pathogenesis remains unclear.^1^ Recent evidence implicates that the intestinal epithelial barrier which consists of intestinal epithelial cells (IECs) and intact tight junction is essential for maintaining the intestinal homeostasis, and the dysfunction of intestinal epithelial barrier contributes to different intestinal diseases, such as IBD.^2^ Although a panel of crucial candidate IBD-susceptibility genes associated with the IBD pathogenesis has been identified in the genetic field,^3^ the regulation mechanism, in particular post-transcriptional modification level of those genes, has not been focused on.

Alternative splicing (AS) is a process of post-transcriptional modification and regarded as a key regulation mode for gene expression and proteomic diversity, producing variants of messenger RNA (mRNA) and proteins from limited mammalian genes, which are thought to perform different biological functions.^4,5^ In general, according to the location of splicing sites in exon or intron segments, AS events can be divided into the following 5 types: alternative 5ʹ splice site (A5SS), skipped exon (SE), alternative 3ʹ splice site (A3SS), retained intron (RI), and mutually exclusive exons (MXEs).^6^

Alternative splicing is implicated in many biological processes and diseases. For example, AS significantly contributes to tissue development and cell differentiation, including brain and heart development, T-cell activation, myoblast, and smooth muscle cell differentiation.^7^ Dysregulation of AS significantly leads to muscular dystrophy,^8^ Alzheimer’s disease,^9^ and colorectal cancer.^10^ The microarray analysis of mucosal tissue of IBD patients has identified 33 differential AS events of intron retention and 47 different expression splicing factors, which are shared by CD and UC in the majority cases.^11^ The transcriptome profiling of colonic biopsies from long- and short-duration UC patients indicates that a mass of genes has differential AS events, which underscores the relationship between AS and IBD.^12^ Therefore, exploring the post-transcriptional modification mechanisms in IECs is of importance not only to deeply understand the progression of IBD but also to develop new targeted therapies of clinical interest.

Considering the importance of IECs to the intestinal barrier, in order to make clear the genome-wide AS profiling in IECs during IBD process, in this study, we focused on the IEC-related global pre-mRNA AS profiling of dextran sodium sulfate (DSS)-induced acute colitis by RNA sequence (RNA-seq). The top differently expressed AS events were verified by reverse transcription polymerase chain reaction. Gene ontology (GO) analysis and Kyoto Encyclopedia of Genes and Genomes (KEGG) enrichment analysis were performed. The results proved that there was a correlation between AS events in IECs and IBD.

## MATERIALS AND METHODS

### Animal Models

The male C57BL/6 mice (approximately 20 g) were purchased from TengXin Biotechnology Corporation (Chongqing, China). The mice provided with drinking water were used as the control group. The acute colitis was induced by 3% DSS in drinking water for 7 days, as previously described.^13^ Each group contains 10 mice. All mice were euthanized by cervical dislocation after isoflurane anesthesia, and then the colon was collected for IEC isolation. All procedures above were acted in accordance with the rule of Laboratory Animal Welfare and Ethics Committee of Army Medical University (SYXK 20170002).

### Isolation of Intestinal Epithelial Cells

The method of IEC isolation was described in our previous study.^14,15^ Firstly, the whole colon tissue was collected and gently washed in Roswell Park Memorial Institute (RPMI) culture medium (Procell, Wuhan, China), supplied with 10% bovine serum (Every Green, Huzhou, China). Secondly, the colon was cut into pieces of approximately 5 mm and thoroughly rinsed with ice-cold phosphate-buffered solution (PBS) with 2% serum. Then, the rinsed colon pieces were incubated in digestive medium (PBS containing 10% serum, 2 mM dithiothreitol, and 5 mM ethylenediaminetetraacetic acid) for 30 minutes at 37°C with continuous violent shaking. Afterward, the supernatant was transferred onto 70 μM and 30 μM Smart Strainers (MACS, Bergisch Gladbach, Germany) to remove impurity and cell aggregate and then centrifuged for collecting cells. The IECs were isolated by centrifuging in 40% Percoll (GE Healthcare, Chicago, Illinois, USA).

### RNA Extraction and Sequencing

The purified IECs were collected, and total RNAs were extracted using RNAiso Plus (Takara, Kusatsu, Shiga, Japan). Then, the RNA concentration and purity of each sample were determined, and the integrity was assessed. The acute colitis group had 6 biological replicates (3 for control and 3 for DSS colitis) for RNA-seq.

The RNA library was established using VAHTS Total RNA-seq Library Prep Kit (Vazyme, Nanjing, China), as described in our previous study.^14^ Briefly, the ribosomal RNA was removed, and the RNA was fragmented into little pieces after being purified. Then, the RNA was transcribed to complementary DNA (cDNA) in 2 steps. After that, these cDNA fragments were added with an “A” base in the terminal and then ligated to the adapters. Finally, the cDNA library was acquired through PCR amplification of these ligated products after being purified. The purified library was quantified and the size distribution was validated. At last, the cDNA library was sequenced using Illumina NovaSeq 6000 by Sinotech Genomics Corporation (Shanghai, China).

All of the output sequence files were quality controlled and then aligned with the mmu GRCm38.91 genome sequences using Hisat2 v2.0.5, and the converted binary alignment/map (BAM) files were obtained. The expression level of all transcripts was calculated by fragments per kilobase of exon per million reads mapped (FPKM). The RNA-seq data were uploaded to Sequence Read Archive (SRA) of NCBI (SRA accession: PRJNA637224).

### Alternative Splicing Analysis

The replicate Multivariate Analysis of Transcript Splicing (rMATS) was used to detect differential AS of paired replicates between different sample groups.^6^ In the rMATS program, the AS events were classified into 5 types, including SE, A5SS, A3SS, MXE, and RI. In this study, the threshold of |Inclevel Differences|>5% and false discovery rate (FDR) < 0.05 were used to assess the statistically significant differential AS events between control and DSS-induced colitis groups.

### Gene Ontology Analysis and Kyoto Encyclopedia of Genes and Genomes Pathway Enrichment

The lists of genes with significantly differential AS events were submitted to the DAVID Bioinformatics Resources 6.8 database for gene functional classification. The GO terms of biological processes, molecular function, and cellular components were analyzed independently, and the KEGG pathway enrichment was performed.

### Polymerase Chain Reaction Validation

The total RNA extracted from IECs was reverse transcribed to cDNA using 2-step RT Reagent Kit (Takara). Candidate AS events were validated by PCR with the primers in Table 1. In detail, cDNA was amplified using Premix Ex Taq (Takara) with the following PCR procedures: 98°C for 5 minutes, 30 cycles of 98°C for 10 seconds, 55°C for 30 seconds, and 72°C for 30 seconds. The PCR product was subjected to 1.5% agarose gel electrophoresis.

### Statistical Analysis

One-way analysis of variance was used for statistical analysis of control and DSS groups, followed by Tukey’s multiple-comparison post hoc tests. A *t*-test was employed to determine *P*-values that were adjusted by FDR. In GO analysis and the KEGG pathway analysis, *P* < .05 was used as the statistically significant criterion.

## RESULTS

### Alternative Splicing Event Profiling in Intestinal Epithelial Cells of Dextran Sodium Sulfate-Induced Colitis

**Figure 1.**
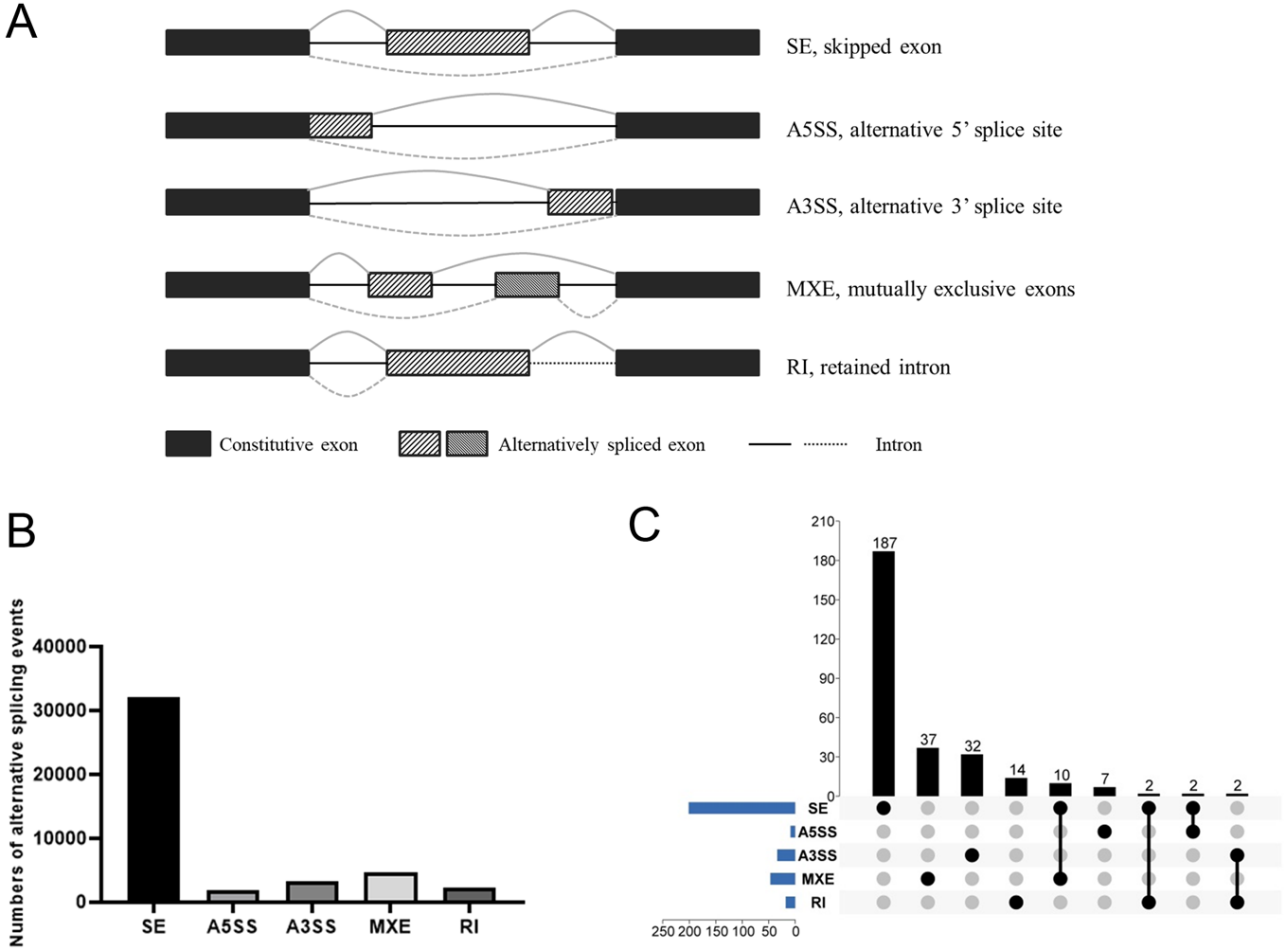
Overview of AS events profiling. (A) Overview of the AS type; (B) The total AS number of acute models; (C) The UpSet plot for difference AS events in IECs of acute colitis model (n = 340, FDR < 0.05, |Inclevel Differences|>5%). AS, alternative splicing; FDR, false discovery rate; IEC, intestinal epithelial cell.

Five types of AS events (A3SS, A5SS, MXE, RI, and SE) were identified in this study using rMATS, with only-water-supplied mice group as the control group (Figure 1A). Totally, 44 474 AS events were identified from IECs of acute colitis. The number of SE type was highest for 32 139 in IECs of acute colitis; thus, SE became the most prevalent AS type in IECs of DSS-induced colitis (Figure 1B).

Directly comparing the significantly differential AS events expression is an effective approach to screen vital roles that involved in the corresponding biological process of colitis. Thus, the FDR <0.05 and |Inclevel Differences|>5% were used to screen the significantly differential AS events between colitis and normal tissues. In IECs of acute colitis, a total of 340 differential AS events were preliminarily screened from 293 genes. Among this, there were 228 SE (corresponding to 201 genes), 9 A5SS (9 genes), 34 A3SS (34 genes), 51 MXE (47 genes), and 18 RI (18 genes) (Figure 1C). In view of this, the UpSet plot was constructed to visualize the intersecting sets of each AS type (Figure 1C). Most AS events were from 1 gene, whereas 1 gene may have up to 4 different types of AS events. Considering SE was the most enriched AS type, top 10 differential AS events of SE type in IECs of acute colitis are listed in Table 2.

### Validation of the Splicing Events in Colitis

**Figure 2.**
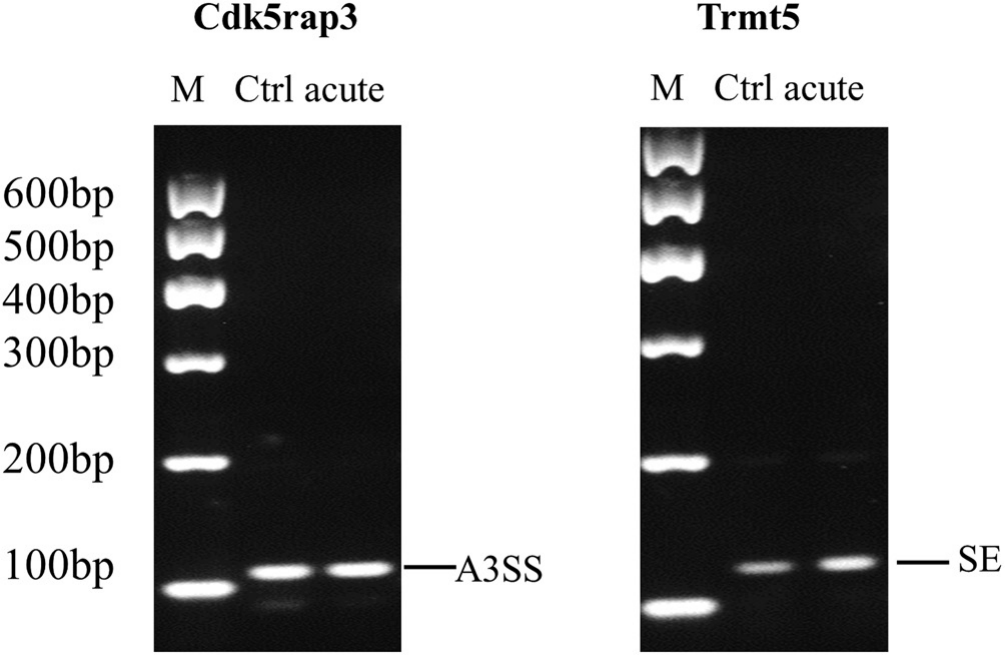
Validation of the top AS events in acute colitis models. AS events validated by polymerase chain reaction in acute colitis model; The primers are listed in Table 1. The types and the products of AS were labeled. A3SS, alternative 3ʹ splice site; AS, alternative splicing; Cdk5rap3, CDK5-regulatory subunit associated protein 3; SE, skipped exon; Trmt5, TRM5 tRNA methyltransferase 5.

Polymerase chain reaction and agarose gel electrophoresis were performed to validate their splicing and expression. As shown in Figure 2, the AS events of CDK5-regulatory subunit associated protein 3 (Cdk5rap3) and TRM5 tRNA methyltransferase 5 (Trmts) in acute colitis model were consistent with the rMATS results.

### Functional Enrichment Analysis of Differential Alternative Splicing Events

**Figure 3.**
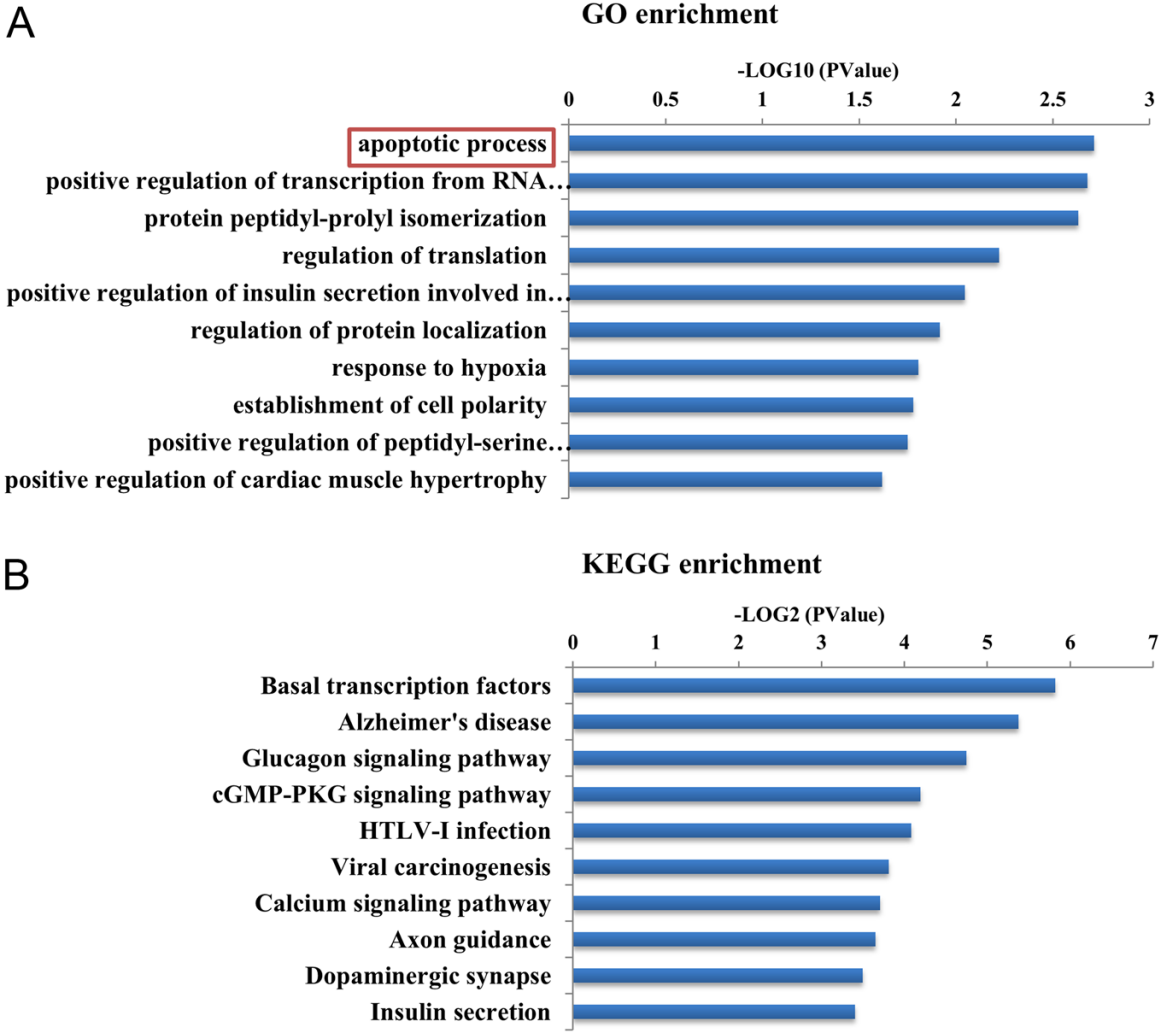
Functional analyses of gene ontology (GO) and Kyoto Encyclopedia of Genes and Genome (KEGG) enrichment of differential alternative splicing events in acute colitis. (A) Gene ontology term enrichment of biological process in acute colitis models and (B) KEGG terms enrichment of acute colitis models. The top 10 biological processes and KEGG pathways are listed.

There were a relatively larger number of differential AS events between colitis and control. In order to analyze the potential functions of those AS events involved in colitis, genes undergoing differential AS events from IECs of acute colitis compared with control, DAVID bioinformatics analysis (DAVID 6.8) was performed. Gene ontology analysis showed the top biological processes in IECs of acute colitis model, such as apoptotic process, positive regulation of insulin secretion involved in cellular response to glucose stimulus, response to hypoxia, establishment of cell polarity, and so on (Figure 3A).

Furthermore, the KEGG pathway analysis showed the pathway distribution of the acute colitis model. Differential AS events from IECs of acute colitis were enriched in Alzheimer’s disease, glucagon-signaling pathway, cyclic guanosine-3’,5’-monophosphate (cGMP)- protein kinase G (PKG)-signaling pathway, Human T-cell leukemia virus type 1 (HTLV-I) infection, calcium-signaling pathway, insulin secretion, and so on (Figure 3B).

**Figure 4.**
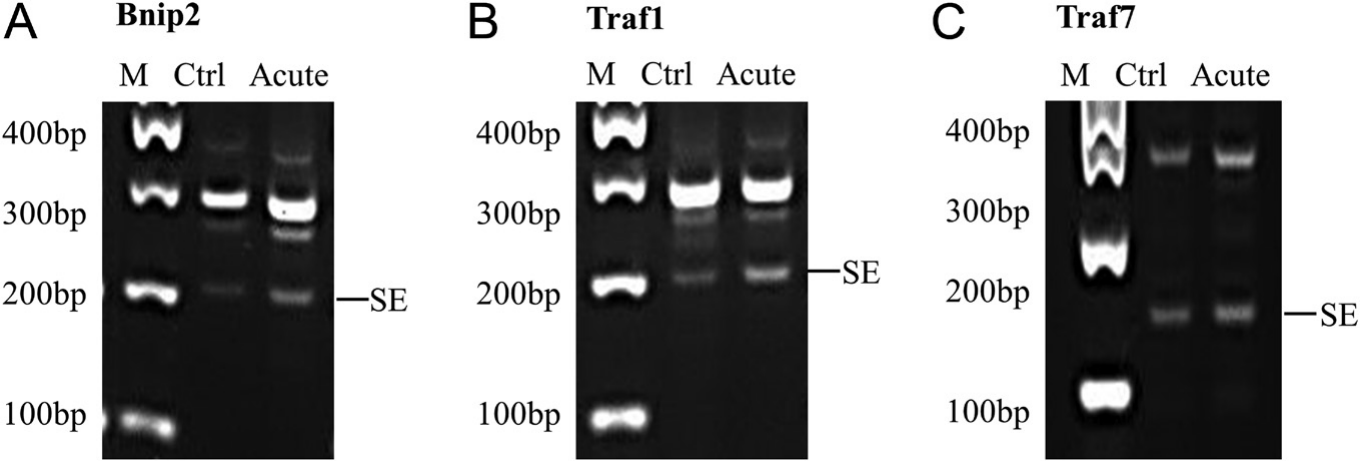
Validation of AS events in apoptotic process of acute colitis models. (A–C) Polymerase chain reaction validation of AS events in acute colitis. The primers are listed in Table 1. The types and the products of AS were labeled. AS, alternative splicing; Bnip2, BCL2/adenovirus E1B-interacting protein 2; Traf1, tumor necrosis factor receptor-associated factor 1; Traf7, tumor necrosis factor receptor-associated factor 7.

The GO analysis showed that the B-cell chronic lymphocytic leukemia/lymphoma 2 (BCL2)/adenovirus E1B 19 kDa-interacting protein 2 (Bnip2), tumor necrosis factor receptor (TNFR)-associated factor 1 (Traf1), and tumor necrosis factor receptor (TNFR)-associated factor 7 (Traf7) enriched in apoptotic process in acute colitis models. The PCR and agarose gel electrophoresis results confirmed the AS types of Bnip2, Traf1, and Traf7 in acute colitis models (Figure 4).

## DISCUSSION

Alternative splicing, which is regulated by RNA-binding proteins, is tissue specificity and identified by cis-acting splicing sequences.^16^ Alternative splicing studies are mainly used to monitor the mRNA expression at exonic resolution using RNA-seq under different experimental conditions, including knockdown, knockout, or overexpression of condition-specific splicing factors.^17^ Alternative splicing of pre-mRNA has been demonstrated to impact the pathogenesis and progression of inflammatory disease, exemplified by IBD. In the previous study, 47 differently expressed splicing factors have been identified in the colon mucosal tissue of IBD patients compared with the healthy controls, which may subsequently mediate the aberrant splicing of intron retention; thus playing potential roles in the pathogenesis of IBD.^11^ The AS of mRNA also has been reported to associate with adherent microbiota in different tissue types and inflammation stages of IBD.^18^ Furthermore, it has been reported that the epithelial splicing-regulatory protein 1 (ESRP1), which is a regulator on AS mainly in epithelial cells, and maintains the integrity of intestinal epithelial barrier by modulating tight junction and IEC proliferation.^19^ In the current study, we have identified differential AS events in epithelial cells in DSS-induced acute colitis models, which further evidenced that pre-mRNA AS is associated with the inflammation progression of colitis and may play potential roles in the regulation of intestinal epithelial barrier function.

Among all the AS events, the most prevalent splicing types of SE have been enriched. Alternative splicing events have been mainly divided into 5 types, including SE-type, MXE-type, A5SS-type, A3SS-type, and RI-type events.^20^ Skipped exon events resulted from the skipping of alternative exon and are most commonly observed in IECs from colitis in this study. It’s worth noting that AS events of SE-type accounted for 67.06% in IECs of acute colitis. Alternative splicing events of A5SS type almost account for the lowest proportion in IECs of acute colitis.

Excessive IEC death is one characteristic of IBD, leading to the destruction of intestinal epithelial barrier integrity.^21^ Furthermore, the IEC proteome studies in IBD patients and mouse models of colitis revealed the association between apoptosis and IBD.^22^ In this study, GO function analysis of genes with significant differential AS events has also presented that the cell apoptosis is a key biological process affected by AS in IECs of acute colitis. The AS events of Bnip2, Traf1, and Traf7 that enriched in apoptotic process in acute colitis models have been verified by PCR. BCL2/adenovirus E1B-interacting protein 2 is a mediator of antiapoptotic activity, and its increased expression can lead to a large number of cell death.^23^ Moreover, it has been reported that the expression of Traf1 is increased in colonic mucosa of IBD patients.^24^ The hypermethylation of Traf7 is associated with colon cancer.^25^ Tumor necrosis factor receptor-associated factor 7 participated in suppressing the hyperpermeability of endothelial cells in inflammatory response,^26^ which indicates Traf7 may be related to the epithelial barrier of IBD. However, the function about the AS events of these genes in colitis still needs further research.

In this study, huge numbers of RNA population derived from AS contribute to the large sequence pool in IECs of DSS-induced acute colitis by RNA-seq. Specific splicing sites for top 10 differential AS events of SE type in IECs of acute colitis have been identified. These provide novel insights to investigate the potential AS events contributing to intestinal epithelial barrier function in IBD.

## Figures and Tables

**Table 1.** Primers Used for PCR Validation for AS Events

Gene	AS Type	Primers	Products
Cdk5rap3	A3SS	CCATCGACATCCAGACCAG	190/109 bp
		GCAGTGTTGATTTTTTCCC	
Trmt5	SE	TTTCCGAGCTGCACCAGAG	190/118 bp
		GGCACCAGGGTAAACAACA	
Bnip2	SE	GAAACTGATGGCCCCAGACA	286/200 bp
		ACTCAGTAATCGAGCCTTTCCT	
Traf1	SE	CACCTGAAACCCCAAGATG	287/202 bp
		TGAAGGAACAGCCAACACC	
Traf7	SE	CGACCGTCACTACCATCAC	232/140 bp
		GGCTCCTCCTCCTCCTCAG	

A3SS, alternative 3ʹ splice sites; AS, alternative splicing; Bnip2, BCL2/adenovirus E1B-interacting protein 2; CDK5, regulatory subunit-associated protein 3; PCR, polymerase chain reaction; SE, skipped exon; Traf1, tumor necrosis factor receptor-associated factor 1; Traf7, tumor necrosis factor receptor-associated factor 7; Trmts, TRM5 tRNA methyltransferase 5.

**Table 2.** Top 10 Differential AS Events of SE Type in DSS-Induced Acute Colitis

Gene	Variant—Exon	IncLevel Difference		FDR
Upregulated				
Lat2	V201-E7, V205-E6	0.784	0.000	
Zfp934	V203-E4, V202-E3	0.753	0.000	
Lat2	V208-E7	0.734	0.005	
Gclc	V202-E2	0.677	0.010	
Jag2	V201-E10	0.667	0.030	
Gm7854	V202-E4	0.667	0.000	
Cd44	V201-E8	0.612	0.031	
Sh2b3	V202-E2	0.606	0.001	
Mthfsl	V204-E2	0.605	0.038	
Ccdc142	V201-E4	0.601	0.007	
Downregulated				
Trmt5	V203-E2	−0.717	0.003	
Dnajb5	V201-E2	−0.667	0.035	
Akap1	V205-E3	−0.666	0.000	
Syt7	V202-E5	−0.608	0.006	
Zfp346	V203-E6	−0.530	0.013	
Zfp467	V207-E5	−0.526	0.001	
Arfip1	V201-E5, V202-E6, V203-E6	−0.508	0.009	
Rpe	V202-E2, V203-E2	−0.503	0.018	
Gm45397	V201-E2	−0.475	0.011	
Zfp182	V203-E2	−0.416	0.019	

Akap1, A kinase (PRKA) anchor protein 1; Arfip1, ADP-ribosylation factor-interacting protein 1; AS, alternative splicing; Ccdc142, coiled-coil domain containing 142; Cd44, CD44 antigen; Dnajb5, DnaJ heat-shock protein family (Hsp40) member B5; DSS, dextran sodium sulfate; FDR, false discovery rate; Gclc, glutamate–cysteine ligase, catalytic subunit; Gm45397, predicted gene 45397; Gm7854, predicted gene 7854; Jag2, jagged 2; Lat2, linker for activation of T cells family, member 2; Mthfsl, 5,10-methenyltetrahydrofolate synthetase; Rpe, ribulose-5-phosphate-3-epimerase; SE, skipped exon; Sh2b3, SH2B adaptor protein 3; Syt7, synaptotagmin VII; Trmt5, TRM5 tRNA methyltransferase 5; Zfp182, zinc finger protein 182; Zfp346, zinc finger protein 346; Zfp467, zinc finger protein 467; Zfp934, zinc finger protein 934.
